# Supplementary material for: Optimization of differentiation and transcriptomic profile of THP-1 cells into macrophage by PMA
Source: PLoS One. 2023 Jul 17;18(7):e0286056. doi: 10.1371/journal.pone.0286056 (PMC10351730; doi:10.1371/journal.pone.0286056)
Supplement: S1 File — (ZIP) [file pone.0286056.s001.zip › supporting files/RNA-seq report.zip!/RNA-seq report/4_GOEnrichment_result/induce_vs_control/DAG/induce_vs_control.DEG_up_mf_DAG.pdf]

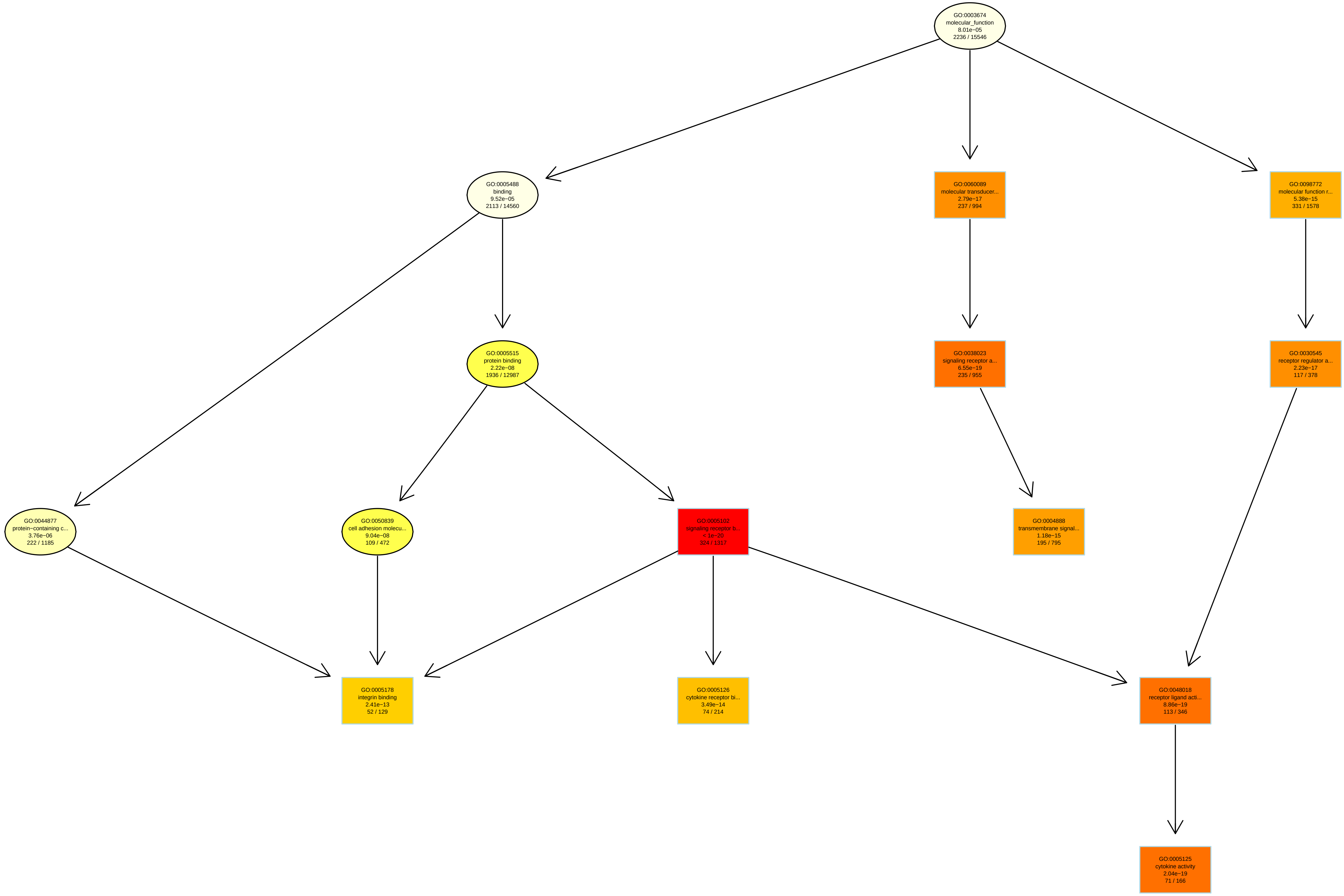
GO:0003674
molecular_function
8.01e−05
2236 / 15546
GO:0005488
binding
9.52e−05
2113 / 14560
GO:0060089
molecular transducer...
2.79e−17
237 / 994
GO:0098772
molecular function r...
5.38e−15
331 / 1578
GO:0005515
protein binding
2.22e−08
1936 / 12987
GO:0038023
signaling receptor a...
6.55e−19
235 / 955
GO:0030545
receptor regulator a...
2.23e−17
117 / 378
GO:0044877
protein−containing c...
3.76e−06
222 / 1185
GO:0050839
cell adhesion molecu...
9.04e−08
109 / 472
GO:0005102
signaling receptor b...
< 1e−20
324 / 1317
GO:0004888
transmembrane signal...
1.18e−15
195 / 795
GO:0005178
integrin binding
2.41e−13
52 / 129
GO:0005126
cytokine receptor bi...
3.49e−14
74 / 214
GO:0048018
receptor ligand acti...
8.86e−19
113 / 346
GO:0005125
cytokine activity
2.04e−19
71 / 166
